# Daily sitting time and past-year falls in Japanese adults: an exploratory cross-sectional analysis

**DOI:** 10.3389/fspor.2025.1610010

**Published:** 2025-09-03

**Authors:** Shigekazu Ukawa, Yusuke Kato, Yonggeun Lee, Masaaki Sugiyama, Hiroko Saito, Kazuoki Ohara, Kazuhiko Mori

**Affiliations:** 1 Osaka Metropolitan University Graduate School of Human Life and Ecology, Osaka, Japan; 2 Kinjo Gakuin University College of Human Life and Environment, Aichi, Japan; 3 The University of Tokyo Graduate School of Engineering, Tokyo, Japan; 4 Osaka Metropolitan University Urban Resilience Research Center, Osaka, Japan; 5 Yokohama City University Graduate School of Urban Social and Cultural Studies, Kanagawa, Japan; 6 Yokohama National University Faculty of Urban Innovation, Kanagawa, Japan

**Keywords:** falls, sedentary behavior, sitting time, ROC curve, Japan, cross-sectional study

## Abstract

**Background:**

Sedentary behavior is a potentially modifiable risk factor for falls, yet the sitting time threshold linked to elevated fall risk remains unclear, particularly in Asian populations.

**Objective:**

This study aimed (1) to examine the association between dichotomized daily sitting time and falls in the past year, and (2) to determine an optimal threshold above which the odds of falls significantly increase.

**Methods:**

A cross-sectional survey was conducted in 2022 in two aging Japanese communities. Data were collected from 1,331 adults (mean age 68.3 ± 15.2 years; 52.1% women) via self-administered questionnaires. The exposure was self-reported daily sitting time from the Global Physical Activity Questionnaire, and the outcome was at least one fall in the past year. Receiver operating characteristic (ROC) analysis identified the optimal threshold, and logistic regression estimated odds ratios (ORs).

**Results:**

Among 1,331 adults, 20.2% reported ≥1 fall in the past year. ROC analysis identified 4.08 h/day as the optimal cutoff (AUC = 0.52). In adjusted models, sitting ≥4.08 h/day was associated with higher odds of falls (crude OR = 1.44, 95% CI: 1.09–1.91; adjusted OR = 1.35, 95% CI: 1.01–1.81). Continuous sitting time was also positively associated with falls.

**Conclusion:**

Daily sitting time of about 4 h or more was linked to increased odds of past-year falls, but its discriminatory ability was minimal (AUC ≈ 0.5). Adding measures such as muscle strength and balance may improve prediction, and this cutoff could serve as a population-level marker when combined with other risk indicators.

## Introduction

Falls are a significant global public health concern across the adult lifespan, affecting not only older adults but also middle-aged and younger adults. They are among the leading causes of injury-related morbidity and mortality, with consequences ranging from minor injuries to fractures and head trauma requiring medical attention (1–3). While the risk is highest in older adults, approximately one-third of community-dwelling adults aged 65 years or older experience at least one fall annually (2). Falls in mid-life can also impose substantial health and economic burdens, especially when compounded by comorbidities or occupational hazards. Preventing falls is therefore a public health priority for all adult age groups.

Sedentary behavior is defined as any waking activity performed while sitting or reclining with an energy expenditure of 1.5 METs or less (4). Objective assessments indicate that adults typically accumulate approximately 8 to 12 h per day in sedentary behavior (5). Prolonged sedentary behavior negatively impacts physical health, particularly traits related to physical frailty, such as bone density and muscle strength, potentially increasing fall risk across the adult lifespan (6). Longitudinal studies involving adult populations have demonstrated that prolonged periods of uninterrupted sitting are associated with reduced muscle strength and accelerated bone loss at the spine (7–9).

Observational and epidemiological studies suggest that greater sedentary time is associated with an increased likelihood of falls across adult populations (10). Cross-sectional and population-based studies report that adults with higher sedentary behavior have approximately 1.2 to 1.3 times greater odds of experiencing a fall compared to those who are less sedentary, even after adjusting for key demographic and health factors (10). In older adults, several studies have proposed sedentary time thresholds of around 4 h per day, above which fall risk increases (11). In this study, we define older adults as aged 65 years or older, consistent with the Japanese convention; some studies, such as one from Brazil, used a threshold of 60 years, which should be considered when comparing findings across countries (11). A Brazilian study of community-dwelling older adults found that sedentary time exceeding approximately 4 h per day was associated with higher rates of falls, with sensitivity and specificity both around 58% (11). These findings suggest that a sedentary duration of about 4 h per day may represent a threshold at which fall risk increases meaningfully, potentially applicable across a broader adult age range.

Most evidence to date comes from Western populations, and data from Asian countries such as Japan remain limited. Cultural and lifestyle differences may influence the relationship between sedentary behavior and fall risk, suggesting a need for population-specific thresholds (12). In Japan, few studies have examined this association across adult age groups (13). A recent study among middle-aged Japanese women found that sedentary behavior exceeding 7 h per day was associated with increased fall risk (13). However, broader adult populations in Japan, including younger and older adults of both sexes, remain under-researched. To address this gap, we examined the association between dichotomized daily sitting time and history of falls among community-dwelling Japanese adults using cross-sectional data. We first evaluated whether higher sedentary time was associated with greater odds of reporting a fall in the past year, and then used ROC analysis to identify a questionnaire-based sedentary time threshold linked to elevated fall risk. Older adults were defined as aged 65 years or older, consistent with Japanese convention, although some countries, such as Brazil, use a threshold of 60 years (11). We report overall results as well as prespecified analyses stratified by age (<65 and ≥65 years).

## Materials and methods

### Study design and participants

This cross-sectional study was conducted in the first quarter of 2022 and involved the recruitment of 2,452 individuals from two regions in Japan: Area A in Aichi Prefecture and Area B in Kanagawa Prefecture. These regions were specifically chosen because they have higher population aging rates (38.6% in Area A and 45.5% in Area B) compared to the national average of 26.7% (14), which increased the expected number of past-year fall events and thus statistical power for ROC analyses and multivariable logistic regression. All community-dwelling adults, regardless of age, were invited. The sampling frame covered all households in the neighborhoods rather than institutions, enhancing feasibility through partnerships with local councils. Both areas, recognized as new towns (15), face significant challenges related to rapid aging and population decline. To facilitate the study, consent and support were obtained from local neighborhood council chairpersons in the selected regions. Due to the absence of prior detailed resident information, questionnaires were distributed to every household within the designated neighborhoods by community association directors. A reminder was provided to encourage the head of each household to complete the survey, and the directors collected the completed questionnaires one week later. Because no resident roster was available, every household in the two neighborhoods was invited, resulting in a convenience sample rather than random sampling. Participation was entirely voluntary, with no financial or other incentives offered; returning the questionnaire was deemed to signify informed consent. All participants received a written participant information sheet together with the questionnaire. The sheet explained the purpose of the study, the ethical approval status, any potential risks associated with participation, the confidentiality of their data, the name and contact details of the principal investigator, and that the study protocol had been approved by the ethics committee of the institution. All residents living in the selected neighborhoods were eligible for participation. Participants were excluded from the analysis if they had missing data for any of the following key variables: average daily sitting time, history of falls, age, sex, weekly walking or other physical activity, or self-rated health. The study protocol received approval from the Ethics Review Committee of the Osaka Metropolitan University Graduate School of Human Life and Ecology (approval no. 22–53). Of the 1,569 respondents (a 64.0% response rate), participants with missing data on key variables, including average daily sitting time (*n* = 215), history of falls (*n* = 2), age (*n* = 9), sex (*n* = 4), weekly walking or other physical activity (*n* = 4), and self-rated health (*n* = 4), were excluded. Consequently, the final analysis included 1,331 individuals (638 men and 693 women). Eligible respondents were community-dwelling adults; age was recorded for all participants.

### Data collection

Data were gathered using self-administered questionnaires. Average daily sitting time was measured with the Global Physical Activity Questionnaire (GPAQ) (16, 17). Participants were asked, “How much time do you usually spend sitting or reclining on a typical day?” This question captured the total time participants spent sitting or reclining during various activities, such as working, being at home, commuting, or socializing. For example, respondents reported time spent sitting at a desk, with friends, traveling by car, bus, or train, reading, playing cards, or watching television; time spent sleeping was excluded. A history of falls was determined by asking whether participants had experienced any falls in the past 12 months, with a fall defined as an unintentional descent to the ground or a lower level. Additional variables collected included age, sex, weekly walking or other physical activities (yes or no), and self-rated health (very good, fairly good, not very good, or poor).

### Data analysis

Receiver operating characteristic (ROC) curve analysis was conducted to assess the diagnostic accuracy of daily sitting time for predicting a history of falls. Age was treated as a continuous covariate, and we prespecified subgroup analyses by age (<65 vs. ≥65 years) and tested a sedentary time × age interaction. The optimal cutoff value was identified based on the maximum Youden index, calculated as Sensitivity plus Specificity minus 1 (18). The area under the ROC curve (AUC) and its 95% confidence interval (CI) were subsequently computed. Logistic regression analysis was then used to estimate both crude and adjusted odds ratios (ORs) for the association between dichotomized daily sitting time (≥4.08 h/day vs. <4.08 h/day) and dichotomized history of falls (yes/no). Participants below the ROC-derived cutoff served as the reference group. The multivariable model was adjusted for age, sex, weekly walking or other physical activity, and self-rated health. Interaction analyses were performed to examine whether the association between daily sitting time and history of falls varied by age, sex, weekly walking or other physical activities, and self-rated health. For each factor, an interaction term was included in separate logistic regression models adjusted for potential confounders, and the significance of these interactions was evaluated using the Wald test. All statistical analyses were performed using R software version 4.4.4 (19), and statistical significance was set at *p* < 0.05.

## Results

Table 1 shows the baseline characteristics of the study participants stratified by history of falls. Of the 1,331 individuals included in the final analysis, 268 (20.1%) reported a history of falls, while 1,063 (79.9%) did not. Participants with a history of falls were significantly older, with a mean age of 72.7 ± 14.5 years compared to 67.2 ± 15.1 years among those without falls (*p* < 0.001). The proportion of men was similar between groups (47.4% in the falls group vs. 48.1% in the non-falls group; *p* = 0.90). Approximately 65.0% of all participants reported engaging in weekly walking or other physical activity, with no significant difference between groups (61.6% vs. 65.9%; *p* = 0.20). In contrast, self-rated health differed significantly, with only 9.7% of participants with a history of falls rating their health as very good compared to 18.3% in the non-falls group (*p* < 0.001).

**Table 1:** Characteristics of the study participants according to history of falls.

Variables	All participants (*n* = 1,331)	With history of falls (*n* = 268)	Without history of falls (*n* = 1,063)	*p*-value
Age (years), mean ± SD	68.3 ± 15.2	72.7 ± 14.5	67.2 ± 15.1	<0.001
Sex, *n* (%)				
Men	638 (47.9)	127 (47.4)	511 (48.1)	
Women	693 (52.1)	141 (52.6)	552 (51.9)	0.90
Weekly walking or other physical activity, *n* (%)				
Yes	866 (65.0)	165 (61.6)	701 (65.9)	0.20
No	465 (35.0)	103 (38.4)	362 (34.1)	
Self-rated health, *n* (%)				
Very good	221 (16.6)	26 (9.7)	195 (18.3)	<0.001
Fairly good	963 (72.4)	181 (67.5)	782 (73.6)	
Not very good	119 (8.9)	48 (17.9)	71 (6.7)	
Poor	28 (2.1)	13 (4.9)	15 (1.4)	

Values are expressed as numbers (percentages) or means ± standard deviations, as appropriate.

*p*-values were calculated using the chi-square test for categorical variables and the *t*-test for continuous variables.

**Figure 1:**
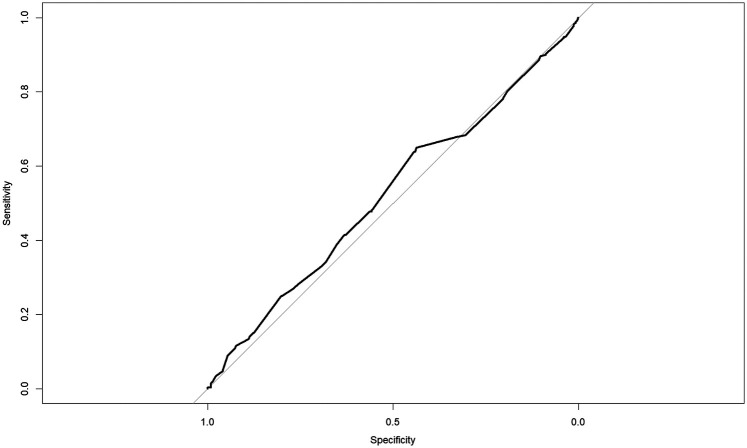
Receiver operating characteristic (ROC) curve for sedentary time predicting history of falls. The solid black line shows the ROC curve (AUC = 0.52).

Table 2 presents the findings from the ROC curve analysis evaluating the diagnostic accuracy of daily sitting time for predicting a history of falls. The optimal cutoff value was determined to be 4.08 h per day based on the maximum Youden index. Figure 1 presents the ROC curve illustrating the predictive ability of sedentary time for history of falls. The curve demonstrates limited discrimination, as reflected by the AUC of 0.52. At this cutoff, sensitivity was 0.65 (95% CI: 0.59–0.71) and specificity was 0.44 (95% CI: 0.41–0.47). The positive predictive value was 0.23 (95% CI: 0.20–0.26), while the negative predictive value was 0.83 (95% CI: 0.80–0.86). The positive likelihood ratio was 1.25 (95% CI: 1.05–1.49) and the negative likelihood ratio was 0.87 (95% CI: 0.78–0.96). Youden's J was calculated as 0.09 (95% CI: 0.04–0.15).

**Table 2:** Diagnostic accuracy of daily sitting time (≥4.08 h/day) in predicting history of falls.

Variables	Cut-off (hours/day)	AUC	Sensitivity	Specificity	+PV	-PV	+LR	-LR	Youden's J
History of falls	≥4.08	0.52 (0.48, 0.56)	0.65 (0.59, 0.71)	0.44 (0.41, 0.47)	0.23 (0.20, 0.26)	0.83 (0.80, 0.86)	1.25 (1.05, 1.49)	0.87 (0.78, 0.96)	0.09 (0.04, 0.15)

ROC, receiver operating characteristic; AUC, area under the curve; +PV, positive predictive value; -PV, negative predictive value; +LR, positive likelihood ratio; -LR, negative likelihood ratio.

Youden's J = Sensitivity + Specificity—1.

Values in parentheses represent 95% confidence intervals.

Table 3 displays the association between daily sitting time and a history of falls. Participants with daily sitting time equal to or exceeding 4.08 h per day had a crude odds ratio (OR) of 1.44 (95% CI: 1.09–1.91) for falls compared to those below the cutoff. After adjusting for age, sex, weekly walking or other physical activity, and self-rated health, the association remained statistically significant, with an adjusted OR of 1.35 (95% CI: 1.01–1.81). No statistically significant interactions were observed for age, sex, weekly walking or other physical activity, or self-rated health.

**Table 3:** Association between daily sitting time and history of falls.

Daily sitting time	Crude OR (95% CI)	Adjusted OR (95% CI)
<4.08 h/day (ref)	1.00	1.00
≥4.08 h/day	1.44 (1.09, 1.91)	1.35 (1.01, 1.81)

OR, odds ratio; CI, confidence interval.

Adjusted for age, sex, weekly walking or other physical activity, and self-rated health.

## Discussion

In this cross-sectional study of Japanese community-dwelling adults, we identified a sedentary time threshold of approximately 4.08 h per day, beyond which the odds of reporting a fall within the past year significantly increased. This finding aligns with previous international studies, suggesting that this threshold may be a common marker of elevated fall risk across diverse populations. Similar values have been reported in Brazilian older adults (11), and greater sedentary leisure time has been associated with increased fall risk among Canadian older adults (10). The consistency of these results across culturally distinct populations suggests that exceeding this duration may be linked to higher fall risk irrespective of sociocultural context. Notably, this threshold is considerably lower than the average sedentary duration of 8 to 12 h per day reported in accelerometer-based studies of older adults (5), indicating that even moderate sitting durations could be relevant for fall prevention. Our sample skewed older (mean age 68.3 ± 15.2 years), which may partly explain the similarity between our cutoff and those reported in older-adult cohorts; therefore, this empirically derived value may primarily reflect the risk profiles of older adults rather than the full adult age range.

Although our findings support a potentially universal sedentary threshold, its implications for falls may vary with cultural and environmental contexts. Routine floor-sitting or squatting is common in some non-Western cultures (20). Sedentary patterns also differ across ethnic groups due to distinct lifestyle and household routines (21). These differences suggest that this threshold may have varying relevance depending on culturally specific physical activities or living environments. Future research should consider such factors to refine fall-prevention guidelines and design strategies suited to diverse populations (22).

Despite the observed association, daily sitting time alone showed poor predictive accuracy for identifying individuals at high fall risk (AUC = 0.52; Youden's index = 0.09; sensitivity = 0.65; specificity = 0.44). These values indicate minimal discrimination and are consistent with previous findings reporting similarly low AUCs (around 0.59) for sedentary time in fall classification (11). The 4.08 h/day threshold should therefore not be used as a standalone screening tool, although it may help stratify risk when combined with other clinical indicators. At the population level, exceeding this threshold identifies increased fall risk, but at the individual level many people above it will not fall, and some below it will, due to other vulnerabilities. Falls result from multiple factors including muscle weakness, impaired balance, gait deficits, and environmental hazards. Sedentary behavior thus represents only one dimension of a complex risk profile (23). Recent meta-analyses support this contributory role, but not as a singular cause, with pooled ORs of approximately 1.17 (1). Incorporating objective physical-function indicators, such as grip strength, timed-up-and-go, and force-plate balance metrics, alongside sedentary time may yield more accurate risk stratification and should be prioritized in future longitudinal studies.

We defined a history of falls as at least one fall in the previous year, consistent with widely used clinical guidelines and screening tools, including the CDC STEADI initiative and the World Guidelines for Falls Prevention and Management. Both recommend using any fall within the past year as a threshold for increased fall risk (22). This definition is intended to maximize the identification of individuals who could benefit from preventive interventions. Nevertheless, we acknowledge that recurrent fallers are at even higher risk for adverse outcomes and that combining single and multiple fallers into one category may limit specificity in risk stratification.

Our study has several limitations that require careful consideration. First, because fall risk increases with age, pooling adults across a wide age range may mask age-specific associations. Given the older age distribution of our sample (mean 68.3 years), generalizability of the ∼4 h/day cutoff to younger adults is uncertain. Although we adjusted for age and tested for interaction, future studies focusing exclusively on community-dwelling older adults are warranted. Second, the cross-sectional design does not permit causal inferences, and the temporal relationship between sedentary behavior and falls cannot be determined. Reverse causation is a legitimate concern, as older adults who have fallen may reduce their activity due to fear of falling and consequently spend more time sitting (24). Longitudinal studies with repeated measurements are necessary to clarify the directionality and predictive value of sedentary behavior as a risk factor for falls. Third, recruitment occurred solely in two Japanese communities with high proportions of older residents, and sampling was non-probabilistic. Although this purposive area selection improved feasibility and event yield for a single-wave survey, it may limit generalizability to younger or nationally representative adult populations. Our sample also included some working-age adults, which may have influenced the characteristics of the study population and introduced selection bias, potentially limiting generalizability to older or retired populations. Fourth, all measurements of exposure and outcome relied on self-report methods. Daily sitting time was collected using a single GPAQ item about a “typical day”, which is prone to recall bias and social desirability bias. Self-reported sedentary behavior often underestimates total sitting time compared with device-based assessments (25), with typical discrepancies of several hours per day. Correlations between questionnaire-based and device-based estimates are generally low (r < 0.25) (26). This systematic underestimation partly explains why the ∼4 h/day threshold identified here is lower than the 8–12 h/day commonly reported in accelerometer-based studies of older adults (5). As a result, our cutoff should be interpreted as a questionnaire-based marker rather than an absolute biological threshold. Falls were also self-reported, and some participants, especially those with minor falls, might not recall every event, potentially biasing the results toward the null or obscuring dose-response relationships. Fifth, although our models adjusted for age, sex, walking or other physical activity, and self-rated health, other important risk factors such as a history of lower extremity fractures or objective measures of balance and mobility were not available. The lack of these variables may have resulted in residual confounding, potentially affecting the estimated association between sedentary time and falls. Future studies should incorporate a broader range of clinical and functional risk factors to better isolate the independent impact of sedentary behavior.

## Conclusion

This study of Japanese community-dwelling adults demonstrated that daily sitting time beyond about 4 h is associated with higher odds of falls. However, sedentary time alone had limited predictive accuracy, highlighting the need for research incorporating objective measurements and additional risk factors to better predict and prevent falls among adults.

## Data Availability

The raw data supporting the conclusions of this article will be made available by the authors, without undue reservation.
