# Correction: Daily sitting time and past-year falls in Japanese adults: an exploratory cross-sectional analysis

**DOI:** 10.3389/fspor.2026.1874514

**Published:** 2026-06-26

**Authors:** Shigekazu Ukawa, Yusuke Kato, Yonggeun Lee, Masaaki Sugiyama, Hiroko Saito, Kazuoki Ohara, Kazuhiko Mori

**Affiliations:** 1 Osaka Metropolitan University Graduate School of Human Life and Ecology, Osaka, Japan; 2 Kinjo Gakuin University College of Human Life and Environment, Aichi, Japan; 3 The University of Tokyo Graduate School of Engineering, Tokyo, Japan; 4 Osaka Metropolitan University Urban Resilience Research Center, Osaka, Japan; 5 Yokohama City University Graduate School of Urban Social and Cultural Studies, Kanagawa, Japan; 6 Yokohama National University Faculty of Urban Innovation, Kanagawa, Japan

**Keywords:** falls, sedentary behavior, sitting time, ROC curve, Japan, cross-sectional study

During the preparation of this work, the authors used Gemini (Google) to reformat the reference list to comply with the journal's submission guidelines. After using this tool, the authors reviewed the reformatted reference list; however, several errors were not identified before publication.

**Reference 1** was erroneously written as Jiang Y, Chang K, Wang Y, Chen Y, Yu P. The association between sedentary behavior and falls in older adults: a systematic review and meta-analysis. *Front Public Health*. (2022) 10:1019551. doi: 10.3389/fpubh.2022.1019551. It should be Jiang Y, Wang M, Liu S, Ya X, Duan G, Wang Z. The association between sedentary behavior and falls in older adults: a systematic review and meta-analysis. *Front Public Health*. (2022) 10:1019551. doi: 10.3389/fpubh.2022.1019551.

**Reference 4** was erroneously written as Tremblay MS, Barnes AS, Saunders JD, Carson TJ, Latimer-Cheung V, Chastin SFM, et al. Sedentary behavior research network (SBRN) — terminology consensus project process and outcome. *Int J Behav Nutr Phys Act*. (2017) 14(1):75. doi: 10.1186/s12966-017-0525-8. It should be Tremblay MS, Aubert S, Barnes JD, Saunders TJ, Carson V, Latimer-Cheung AE, et al. Sedentary behavior research network (SBRN) - terminology consensus project process and outcome. *Int J Behav Nutr Phys Act*. (2017) 14(1):75. doi: 10.1186/s12966-017-0525-8.

**Reference 7** was erroneously written as Gobbo LA, Zanuto EAC, Alexandre TDS, Cirillo MD, de Oliveira C. Sedentary patterns are associated with bone mineral density and physical function in older adults: cross-sectional and prospective data. *Int J Environ Res Public Health*. (2020) 17(21):7902. doi: 10.3390/ijerph17217902. It should be Gobbo LA, Júdice PB, Hetherington-Rauth M, Sardinha LB, Dos Santos VR. Sedentary patterns are associated with bone mineral density and physical function in older adults: cross-sectional and prospective data. *Int J Environ Res Public Health*. (2020) 17(21):8198. doi: 10.3390/ijerph17218198.

**Reference 10** was erroneously written as Lustosa LG, Comim FV, McLeod B, Askin N, Hsu W, Dogra S. Leisure sedentary time is associated with self-reported falls in middle-aged and older females and males: an analysis of the CLSA. *Can Geriatr J*. (2023) 26(2):239–46. doi: 10.5770/cgj.26.642 It should be Lustosa LG, Rudoler D, Theou O, Dogra S. Leisure sedentary time is associated with self-reported falls in middle-aged and older females and males: an analysis of the CLSA. *Can Geriatr J*. (2023) 26(2):239–46. doi: 10.5770/cgj.26.636.

**Reference 13** was erroneously written as Ozaki E, Takayama M, Nishio A, Kitamura K, Sugihara Y, Kobayashi Y. Association between sedentary time and falls among middle-aged women in Japan. *Healthcare*. (2022) 10(12):2565. doi: 10.3390/healthcare10122565. It should be Ozaki E, Matsui D, Kuriyama N, Tomida S, Nukaya Y, Koyama T. Association between sedentary time and falls among middle-aged women in Japan. *Healthcare (Basel)*. (2022) 10(12):2354. doi: 10.3390/healthcare10122354.

**Reference 16** was erroneously written as Keating XD, Zhou K, Liu X, Guan J. Reliability and concurrent validity of global physical activity questionnaire (GPAQ): a systematic review. *Int J Environ Res Public Health*. (2019) 16(21):4128. doi: 10.3390/ijerph16214128. It should be Keating XD, Zhou K, Liu X, Hodges M, Liu J, Guan J, et al. Reliability and concurrent validity of Global Physical Activity Questionnaire (GPAQ): a systematic review. *Int J Environ Res Public Health*. (2019) 16(21):4128. doi: 10.3390/ijerph16214128.

**Reference 20** was erroneously written as Lipsitz LA, Jonsson PV, Kelley MM, Koestner JS, Rowe JW. Causes and correlates of recurrent falls in ambulatory frail elderly. *J Gerontol.* (1991) 46(4):M114–22. doi: 10.1093/geronj/46.4.M114. It should be Mulholland SJ, Wyss UP. Activities of daily living in non-Western cultures: range of motion requirements for hip and knee joint implants. *Int J Rehabil Res*. (2001) 24(3):191–8. doi: 10.1097/00004356-200109000-00004.

**Reference 21** was erroneously written as Kee CC, Rahman MA, Tengku-Aizan H, Shariff ZM, Lim KK, Ghazali E, et al. Sedentary behaviour and its correlates among older adults in Malaysia. *Healthcare*. (2025) 13(2):240. doi: 10.3390/healthcare13020240. It should be Kee CC, Tan LK, Cheah YK, Teh CH, Lim HL, Cheong YL, et al. Sedentary behaviour and its correlates among older adults in Malaysia. *Healthcare (Basel)*. (2025) 13(2):160. doi: 10.3390/healthcare13020160.

**Reference 22** was erroneously written as Montero-Odasso M, van der Velde N, Martin FC, Petrovic M, Ryg J, Iliffe S, et al. World guidelines for falls prevention and management for older adults: a global initiative. *Age Ageing*. (2022) 51(9):afac205. doi: 10.1093/ageing/afac205. It should be Montero-Odasso M, van der Velde N, Martin FC, Petrovic M, Tan MP, Ryg J, et al. World guidelines for falls prevention and management for older adults: a global initiative. *Age Ageing*. (2022) 51(9):afac205. doi: 10.1093/ageing/afac205.

**Reference 23** was erroneously written as Arkkukangas M. Involvement of older adults, the golden resources, as a primary measure for fall prevention. *Clin Interv Aging*. (2023) 18:2165–70. doi: 10.2147/CIA.S428367. It should be Arkkukangas M. Involvement of older adults, the golden resources, as a primary measure for fall prevention. *Clin Interv Aging*. (2023) 18:2165–70. doi: 10.2147/CIA.S430309.

**Reference 25** was erroneously written as Prince SA, LeBlanc AG, Colley RC, Saunders TJ. A comparison of self-reported and device measured sedentary behaviour in adults: a systematic review and meta-analysis. *Int J Behav Nutr Phys Act.* (2020) 17(1):31. doi: 10.1186/s12966-020-00938-3. It should be Prince SA, Cardilli L, Reed JL, Saunders TJ, Kite C, Douillette K, et al. A comparison of self-reported and device measured sedentary behaviour in adults: a systematic review and meta-analysis. *Int J Behav Nutr Phys Act*. (2020) 17(1):31. doi: 10.1186/s12966-020-00938-3.

A correction has been made to the section **Discussion**, paragraph 2:

“ Although our findings support a potentially universal sedentary threshold, its implications for falls may vary with cultural and environmental contexts. Routine floor-sitting or squatting is common in some non-Western cultures (20). Sedentary patterns also differ across ethnic groups due to distinct lifestyle and household routines (21). These differences suggest that this threshold may have varying relevance depending on culturally specific physical activities or living environments. Future research should consider such factors to refine fall-prevention guidelines and design strategies suited to diverse populations (22).”

An incorrect **Generative AI statement** was provided. The correct statement reads:

“The author(s) declare that Generative AI was used in the creation of this manuscript. During the preparation of this work, the authors used Gemini (Google) to reformat the reference list to comply with the journal's submission guidelines. The authors take full responsibility for the content of the publication.

Any alternative text (alt text) provided alongside figures in this article has been generated by Frontiers with the support of artificial intelligence and reasonable efforts have been made to ensure accuracy, including review by the authors wherever possible. If you identify any issues, please contact us.”

The original version of this article has been updated.

